# Retinal capillary hemangioma in a pthisical globe: Late sequelae in a case of Von Hippel-Lindau (VHL) disease presenting with endophthalmitis

**DOI:** 10.1016/j.ijscr.2025.110867

**Published:** 2025-01-12

**Authors:** Hala A. Helmi, Rakan Alsaad, Hattan Alkhiary, Hind M. Alkatan

**Affiliations:** a Department of Ophthalmology, McGill University, Montreal, Canada; b Department of Ophthalmology, King Faisal Specialist Hospital and Research Center, Riyadh, Saudi Arabia; c Department of Ophthalmology, College of Medicine, King Saud University, Riyadh, Saudi Arabia; d King Saud University Medical City, King Saud University, Riyadh, Saudi Arabia; e Department of Pathology and Laboratory Medicine, College of Medicine, King Saud University, Riyadh, Saudi Arabia

**Keywords:** Von Hippel-Lindau, Keratitis, Endophthalmitis, Retinal capillary hemangioma, Hemangioblastoma, Case report

## Abstract

**Introduction:**

Retinal capillary hemangioma (RCH) is a benign vascular hamartoma that can occur sporadically or as a manifestation of Von Hippel-Lindau (VHL) disease. If left untreated, it results in adverse ocular complications depending on its location and eventual visual loss.

**Case presentation:**

We present a 50-year-old man who was a known case of VHL with history of left eye vision loss in the left eye at the age of 30 years. He underwent resection of a brain hemangioblastoma 10 years earlier, which was complicated by left facial nerve palsy. He presented with acute left eye pain, redness, and eyelid swelling. He had large corneal infiltrate with hypopyon and obscured fundus view. He was treated medically as a case of left endophthalmitis with no improvement. The eye was eventually eviscerated. Histopathological examination revealed acute necrotizing keratitis, osseous metaplasia, and long-standing RCH.

**Discussion:**

Peripheral RCHs are the most encountered ocular vascular lesion in VHL disease. Other locations close to the optic nerve (juxtapapillary) result in complicated visual loss. The RCH in our VHL case was confirmed 20 years after the history of vision loss in the same eye. The patient also had confirmed surgically treated intracranial hemangioblastoma but didn't seek any ophthalmic care prior to his recent presentation with painful acute keratitis and endophthalmitis.

**Conclusion:**

VHL has multiple organ involvement. Patients suspected or diagnosed with VHL should receive prompt health care counselling to ensure periodic eye examination for control of any intraocular vascular lesions to prevent visual loss.

## Highlights

• Von Hippel-Lindau (VHL) disease affects several organs including the eye.
• The main ocular lesion in VHL is Retinal capillary hemangioma (RCH).
• We report a 50-year-old man with VHL disease and neglected left eye blindness for 20 years.
• His recent presentation with endophthalmitis lead to evisceration and confirmation of left eye RCH.
• This complicated ocular course of the disease can be prevented by careful periodic eye examination.

## 1 Introduction

Retinal capillary hemangioma (RCH) is one of the early lesions found in about 60 % of patients who are known to suffer from Von Hippel-Lindau (VHL) disease, with bilateral occurrence in half of these cases [1]. Other manifestations of VHL include intracranial hemangiomas and visceral neoplasms affecting other organs such as the adrenal glands, pancreas, and kidneys [2].

RCH represent a benign vascular hamartoma that can slowly grow, causing complicated subretinal exudation and exudative retinal detachment with potential loss of vision, especially if it is juxtapapillary in location or on the optic nerve head itself [[3], [4], [5]]. Peripheral RCHs are the vascular lesions most commonly found intraocularly in association with VHL, but other locations in relation to the optic nerve (juxtapapillary) have been also observed [3,6]. Late ocular changes including osseous metaplasia in affected eyes harboring RCH have been well demonstrated in a previously published case [6]. In this report we similarly present the late ocular histopathological findings in an eye with RCH but in the context of microbial keratitis and early endophthalmitis necessitating evisceration of the eye contents. This case report has been prepared and reported in line with the SCARE criteria [7].

## 2 Presentation of case

A 50-year-old man presented to our ophthalmology emergency department with acute onset of pain and eyelid swelling in his non-seeing left eye over the course of 2 days. This was also associated with redness and corneal whitening. There was no history of recent craniofacial trauma. His past medical history was remarkable for Von Hippel-Lindau (VHL) disease, diagnosed 12 years prior, after findings consistent with brain hemangioblastomas. He underwent a craniotomy for the hemangioblastoma resection with placement of a ventriculoperitoneal shunt 1 year after diagnosis, which was complicated by left facial nerve palsy. He didn't provide clear family history of VHL. The history of loss of vision in the affected eye goes back to 20 years ago but he did not seek medical attention at that time and his loss of vision was attributed later to the complicated ocular manifestations of his systemic disease. Three months prior to his ophthalmic complaint, the patient was suspected to have bilateral renal cell carcinoma and has been undergoing thorough evaluations since then. We had no information about the status of his investigations at the time of writing this case report.

On examination, his vital signs were normal, and his best corrected visual acuity was 20/20 in the right eye and absent light perception in the left eye. His intraocular pressure was 17 mmHg in bilaterally. Extraocular movements were full in all gazes with no restrictions bilaterally. Eyelid swelling, erythema, and tenderness was present in the left eye along with complete lagophthalmos and reduced corneal sensation, which has attributed to the exposure keratopathy and corneal infection. The left eye conjunctiva was severely injected, and the cornea had a large (12 × 8 mm) opaque infiltrate with inferior thinning. A hypopyon involving more than half of the anterior chamber was present in the left eye with no view to the fundus. Anterior and posterior segments examination of the right eye were unremarkable. There was no evidence of any ocular manifestations of VHL in his right eye.

A clinical diagnosis of left eye endophthalmitis secondary to microbial keratitis was made considering the presence of a large infected corneal ulcer and the absence of another endogenous source for his intraocular infection. The patient was admitted aiming to control the severe infection and ordered topical drops of vancomycin and ceftazidime along with intravenous vancomycin and ceftriaxone for 3 days without improvement. Considering the lack of response in a non-seeing eye and the worsening of his symptoms, the treatment options and poor outcome were discussed with the patient, who was mostly concerned about his acute symptoms and evisceration was agreed upon. He underwent the evisceration of the left eye successfully as planned, was fitted with a sphere implant and the intraocular contents were sent for histopathological evaluation.

**Fig. 1:**
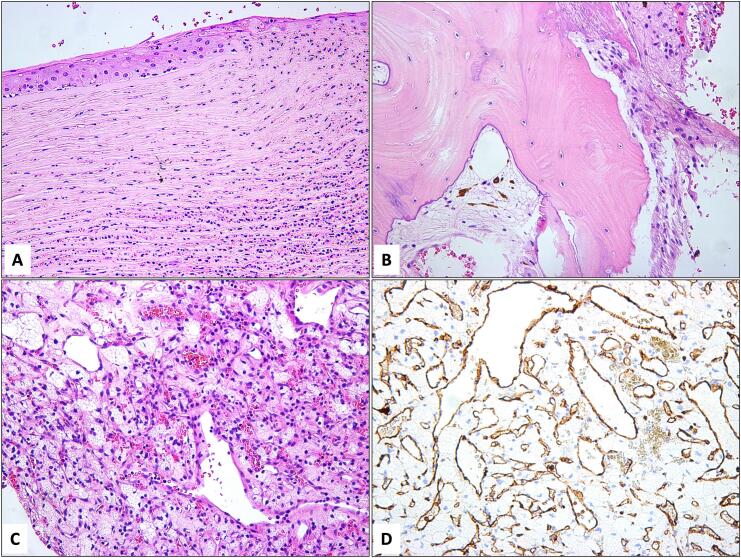
A: The histopathological appearance of the left eye corneal ulcer and acute keratitis with stromal infiltrate by acute polymorphonuclear cells (Original magnification x400 Hematoxylin & Eosin). B: The evisceration tissue showing areas of bone formation as part of phthisis bulbi changes (Original magnification x200 Hematoxylin & Eosin). C: The area of capillary proliferation within the retina and surrounding foamy histiocytes confirming the intraocular retinal capillary hemangioma (Original magnification x200 Hematoxylin & Eosin). D: The outline of the capillaries in the retinal hemangioma using endothelial cell marker (Original magnification x200 CD31).

Histopathological studies revealed thick and irregular corneal epithelium and stroma with acute inflammatory cells, an adjacent area of necrosis, and an area of ulceration (Fig. 1A). The intraocular contents showed a cataractous lens and disrupted uveal tissue. However, adjacent to the choroid, large areas of fibrous tissue and bone formation were observed, representing fibrous and osseous metaplasia of the retinal pigment epithelium (RPE) (Fig. 1B). One focal area in the retina showed marked disruption and gliosis with a large vascular lesion consisting of retinal capillary blood vessels proliferation (Fig. 1C). The capillaries of the vascular lesion were outlined with immunohistochemical staining and expression of CD31 (Fig. 1D). A diagnosis of retinal capillary hemangioma was confirmed as one of the manifestations of the patient's systemic VHL disease that has been untreated thus being the most likely cause for his asymptomatic blindness because of anticipated relevant complications.

The patient recovered uneventfully and was later fitted with an ocular prosthesis. After a couple of postoperative follow up visits, a lateral tarsal strip procedure was performed on the left eye to address the patient's lower eyelid laxity and to stabilize his ocular prosthesis in place. The patient has been stable across his ophthalmic follow up visits for 3 months and was advised to continue the evaluation and work up of his suspected bilateral renal cell carcinoma appointments. Patient's general counselling about his systemic disease was recommended to be provided in the general hospital, where he was to continue his follow up.

**Supplementary Fig. 2:**
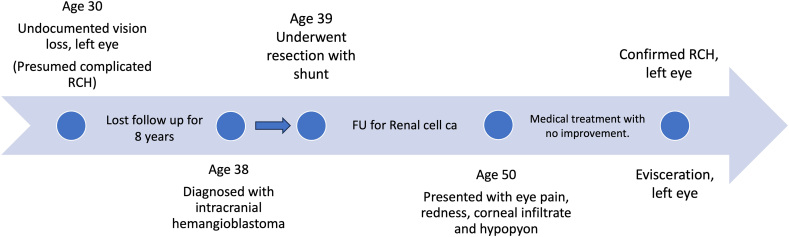
Timeline describing the steps of the patient's presentation and diagnosis. Retinal Capillary Hemangioma in a Pthisical Globe: Late sequelae in a case of Von Hippel-Lindau (VHL) disease presenting with endophthalmitis.

The timeline clarifying the clinical course of his VHL disease is outlined as a Supplementary Fig. 2.

## 3 Discussion

VHL disease is an autosomal dominant disorder resulting from chromosome 3 mutation of the VHL gene on (3p25–26). This results in several organ manifestations and associated vascular lesions that might affect the globe [8]. The risk for development of RCH increases with advancing age [9]. This might be evident in our patient who although was diagnosed with the RCH at the age of 50, he did note the loss of vision in that eye from 20 years prior. This may indicate an earlier development of the RCH around the age of 30 years. Unfortunately, he did not have a documented ocular examination prior to this most recent presentation to us. These lesions my result in several ocular complications such as macular edema and intraretinal and/or subretinal exudation leading to exudative retinal detachment [[4], [5], [6]]. Regarding the RCH location, it is worth mentioning that in our case, the lesion was observed among the examined intraocular contents collected by evisceration rather than enucleation, which makes it difficult for us to confirm the exact primary location of the vascular lesion in relation to the optic nerve. Diagnosis of VHL disease is mostly clinical but genetic testing might be required [2]. Our patient had confirmed VHL disease with diagnosed intracranial hemangioblastoma that was surgically resected, in addition to suspected bilateral renal cell carcinoma. Patients with VHL should have annual ocular examination with dilated fundus examination to diagnose any new vascular lesions and observe progressive changes in existing RCHs with recommended screening protocols that should be implemented lifelong [10,11]. Our patient did not seek any ophthalmic medical attention when he lost the vision in the left eye 20 years prior to presentation. Several local management options might have altered the outcome of his RCH. In general, treatment modalities including observation will depend on the location, size, and progression of the lesion. Ablation therapy is recommended for small early lesions using argon laser photocoagulation with or without cryotherapy [11,12]. Photodynamic therapy, external beam radiation, and surgical therapy in advanced lesions have been also described [11,13]. Unfortunately, he developed left facial nerve palsy and left microbial keratitis, for which he eventually underwent left eye evisceration.

RCHs typically show capillary-like vascular channels histopathologically with surrounding foamy histiocytes [14]. The intraocular contents of the eviscerated globe in our patient apart from the acute necrotizing keratitis, showed confirmation of the retinal vascular lesion, and other features that can be explained by the chronicity of this RCH in a non-seeing eye like the presence of cataract and bone formation [6]. Owing to the complexity of this disease with possible multiple organ involvement, multidisciplinary management including counselling of patients have been recommended [15]. In our case, earlier diagnosis of the RCH would have possibly led to earlier detection of the cerebral hemangioma that are mostly associated with the ocular involvement in VHL disease. In these cases, the gold standard investigation would have been magnetic resonance imaging (with contrast) or cerebral computed tomography scan [15,16]. In addition, genetic testing for mutation of the VHL tumor suppressor gene on chromosome 3p25–26 would have been useful in our case in view of the suspected renal cell carcinoma because of the correlation between the VHL gene alteration and the phenotype aggressiveness of the carcinoma [11,17].

## 4 Conclusion

In conclusion, our case further demonstrates the late histopathological sequelae of neglected RCH in the context of VHL disease resulting in initial blindness then further complicated disease course related to the systemic VHL and the surgical management of his brain hemangioblastoma. The health awareness of this blinding yet treatable disease is relatively low among our general population. Ophthalmologists play a major role not only in diagnosing and controlling the ocular manifestations but also in recommending lifelong surveillance for such patients. General practitioners on the other hand should also contribute to the early recognition of VHL with immediate ophthalmic screening referrals and recommendations for monitoring of the disease.

The following os the supplementary data related to this article.

## Authors' contributions

**Hala A. Helmi & Rakan Alsaad**: Acquisition of data and drafting of the manuscript. **Hattan Alkhiary**: Concept and design of the case report. **Hind M. Alkatan**: Histopathological diagnosis; and critical review of the manuscript for submission.

## Consent

Written informed consent was obtained from the patient for publication and any accompanying images. A copy of the written consent is available for review by the Editor-in-Chief of this journal on request.

## Ethical approval

An ethical approval is not required for case reports as per the regulations of the Institutional Review Board, College of Medicine, King Saud University. However, information was obtained and reported in a manner that was compliant with the standards set forth by the Health Insurance Portability and Accountability Act, and the Declaration of Helsinki as amended in 2013.

## Guarantor

Hind M. Alkatan, MD, Professor, College of Medicine, Departments of Ophthalmology & Pathology, King Saud University Medical City, Riyadh, Saudi Arabia. Email: hindkatan@yahoo.com; hkatan@ksu.edu.sa. Tel. No: +966-504492399, Fax No: +966-11-2052740

## Provenance and peer review

Not commissioned, externally peer reviewed.

## Source of funding

This research did not receive any specific grant from funding agencies in the public, commercial, or not-for-profit sectors.

## Research registration

Not applicable.

## Declaration of competing interest

The authors declare that they have no conflicts of interest..
